# Emphasizing the role of Wnt5a protein expression to predict favorable outcome after radical prostatectomy in patients with low-grade prostate cancer

**DOI:** 10.1002/cam4.5

**Published:** 2012-06-04

**Authors:** Azharuddin Sajid Syed Khaja, Lars Egevad, Leszek Helczynski, Peter Wiklund, Tommy Andersson, Anders Bjartell

**Affiliations:** 1 Division of Urological Cancers, Department of Clinical Sciences, Skåne University Hospital, Lund UniversityMalmö, Sweden; 2 Center for Molecular Pathology, Skåne University Hospital, Lund UniversityMalmö, Sweden; 3 Department of Oncology and Pathology, Karolinska InstituteStockholm, Sweden; 4 University and Regional Laboratories Region Skåne, Clinical PathologyMalmö, Sweden; 5 Department of Molecular Medicine and Surgery, Karolinska InstituteStockholm, Sweden; 6 Division of Cell and Experimental Pathology, Department of Laboratory Medicine, Skåne University Hospital, Lund UniversityMalmö, Sweden

**Keywords:** Androgen receptor, biochemical recurrence, prostate cancer, tissue microarray, Wnt5a

## Abstract

Wnt5a, a member of non-canonical wingless-related MMTV integration site family is a secreted glycoprotein that plays important roles in development and disease. Recent studies have shown that Wnt5a protein levels are up-regulated in prostate cancer, but contrasting reports exist on the role of Wnt5a to predict outcome after radical prostatectomy in patients with localized prostate cancer. Our group has recently shown that preserved high protein expression of Wnt5a in prostate cancer is associated with longer relapse-free time after radical prostatectomy. The present tissue microarray study emphasizes the role of Wnt5a protein expression in a different, well-defined, and independent cohort consisting of 312 prostate cancer patients. Kaplan–Meier curves plotted between Wnt5a expression and time to biochemical recurrence revealed that in low-grade prostate cancer, patients with preserved high-Wnt5a protein levels in their tumor cells have a lower risk of recurrence after radical prostatectomy compared to patients with low-Wnt5a protein expression. When Wnt5a protein expression was added to a Cox regression multivariate analysis, both Wnt5a protein expression and surgical margin status independently predict biochemical free survival. Herein we confirm Wnt5a positivity as a prognostic factor and show that preserved overexpression of Wnt5a protein is associated with increased time to biochemical recurrence in localized low-grade prostate cancer patients after radical prostatectomy. Our results emphasize that Wnt5a can be used as a predictive biomarker, and favoring the view of Wnt5a as a future therapeutic target in prostate cancer patients with tumor cells displaying low expression of Wnt5a.

## Introduction

Prostate cancer (PCa) is the second leading cause of cancer related deaths in men in western world [1]. Androgen deprivation therapy has significant but limited effect on hormone dependent PCa, but there is an urgent need to develop new effective therapies against castration-resistant prostate cancer (CRPC). A better understanding of different molecular mechanisms of PCa development and progression may lead to new therapies. Several studies have highlighted the role of androgens and of the androgen receptor (AR) and its interaction with other factors at different cellular levels. Other tissue biomarkers such as Ki-67 [2], VEGF [3]. and bcl-2 [4] have been shown to be aberrantly expressed in PCa and to predict the risk of relapse after radical prostatectomy (RP). Although the altered expression of many biomarkers represents the molecular heterogeneity of PCa, the clinical usefulness of tissue biomarkers has this far been limited [5]. Several biomarkers have failed to show prognostic value when externally validated in an independent cohort.

Recent studies have highlighted the role of Wnt5a in PCa progression and its value to predict outcome of PCa patients undergoing RP. Wnt5a, one of the members of non-canonical Wnt family, is a secreted glycoprotein involved in organ development, tissue orientation, and cell proliferation and migration. It mainly acts through calcium signaling pathway [6], but it can also stimulate canonical signaling affecting β-catenin in presence of frizzled receptor 4 [7]. Because of its involvement in both canonical and non-canonical signaling pathways, dysregulation of Wnt5a within the cell has been associated with development and progression of several cancers. However, whether Wnt5a acts as a tumor-promoting or tumor-suppressing factor remains a debatable question [6]. It cannot be excluded that Wnt5a might play various roles in different types of cancer. In colon cancer [8], neuroblastoma [9], invasive ductal breast carcinomas [10], and leukemias [11], it has been shown to have a tumor-suppressing effect, whereas in gastric cancer [12], melanoma [13], lung cancer [14], and pancreatic cancer [15], it seems to exert oncogenic effects. Dejmek et al. [8] found reduced Wnt5a protein expression in 50% of stage Dukes B colon cancer and patients with reduced Wnt5a protein expression had an adverse outcome. Blanc et al. [9] showed that Wnt5a mRNA expression was reduced in high-risk neuroblastoma and correlated with unfavorable outcome. In invasive ductal breast carcinomas, loss of Wnt5a expression was significantly associated with shorter relapse-free survival [10]. Dejmek et al. [16] observed reduced expression in more than half of the breast cancer patients (56%), and that high protein expression of Wnt5a indicated favorable outcome. Loss of Wnt5a staining in hepatocellular carcinoma was significantly associated with higher tumor stage [17]. On the contrary, in non-small-cell lung cancer, Wnt5a overexpression was associated with short survival [14] and in gastric cancer, increased Wnt5a protein expression correlated with advanced tumor stages and poor survival [12]. Recent studies have uniformly shown that Wnt5a protein levels are up-regulated in PCa compared to benign prostatic tissue [18–20], although the underlying mechanism is not fully understood. However, conflicting reports exist regarding the value of Wnt5a expression to predict recurrence after RP. Yamamoto et al. [20] have shown that overexpression of Wnt5a correlated with high Gleason scores and that patients with high-Wnt5a protein expression have shorter relapse-free survival time compared to patients with low-Wnt5a protein expression. In contrast, our group recently showed that a preserved high expression level of Wnt5a in PCa is associated with longer time to biochemical recurrence (BCR) [18].

These conflicting reports on the association of Wnt5a with outcome in PCa have prompted us to perform a validation study to clarify the role of Wnt5a to predict outcome in PCa patients utilizing an external independent patient cohort. We were able to confirm Wnt5a protein as a prognostic marker in PCa and that patients with high-Wnt5a protein expression had a favorable outcome compared to patients with low-Wnt5a protein expression in low-grade PCa. We further observed that patients with high-Wnt5a protein expression and positive surgical margin status (SMS) had similar time to relapse as patients with low-Wnt5a expression and negative SMS.

## Materials and Methods

### Ethics statement

The study was approved by the ethics committees at the Karolinska University Hospital, Stockholm (2006/4:10) and at IARC, Lyon (06-08) [21].

### Patient material

The patient cohort used in the present study has been described previously [21]. Briefly, it comprises a consecutive series of patients who underwent RP between May 1998 and November 2002 at the Karolinska University Hospital, Stockholm, Sweden. None of the patients received androgen deprivation treatment or radiation therapy prior to RP. Patients' characteristics are summarized in Table 1 together with details on the cohort in our previous study [18]. Biochemical recurrence was defined as two consecutive serum prostate-specific antigen (PSA) values ≥0.2 ng/mL.

**Table 1:** Patients characteristics of the present cohort (Stockholm) and the previously reported Malmö cohort [18]

	Median (IQR) or frequency (%)	
	
	Current TMA (Stockholm) *n* = 262*	Previous TMA (Malmö) *n* = 503
Age at diagnosis (years)	61 (56, 64)	63 (59, 66)
Preoperative PSA (ng/mL)	7.9 (5.5, 12)	7.2 (5, 11)
Clinical Stage		
T1	167 (63%)	181 (43%)**
T2	91 (35%)	233 (55%)
T3	4 (2%)	9 (2%)
Pathological Gleason Score		
Low-grade (≤3+4)	198 (76%)	423 (84%)
High-grade (≥4+3)	64 (24%)	51 (10%)
EPE	119 (45%)	250 (50%)
SVI	33 (13%)	55 (11%)
SMS positive	118 (45%)	259 (52%)
BCR	100 (38%)	106 (27%)#

IQR, Interquartile range; PSA, prostate-specific antigen; EPE, extraprostatic extension; SVI, seminal vesicle invasion; SMS, surgical margin status; BCR, biochemical recurrence.

* Total number of patients with complete follow-up data and evaluable tissue cores.

** Information about clinical stage available from 423 patients.

# Information about BCR from 397 patients.

A tissue microarray (TMA) was constructed from formalin-fixed paraffin-embedded RP specimens as reported earlier [21]. All samples were selected and reviewed by an experienced pathologist and co-author (L. E.). To obtain representative tumor material, three cores per tumor sample were selected, including two cores from the primary (predominant) Gleason pattern and one core from the secondary Gleason pattern. We had access to tumors from 312 patients, with complete clinical follow-up data available from 262 of them.

### Immunohistochemistry and scoring of TMA cores

**Figure 1:**
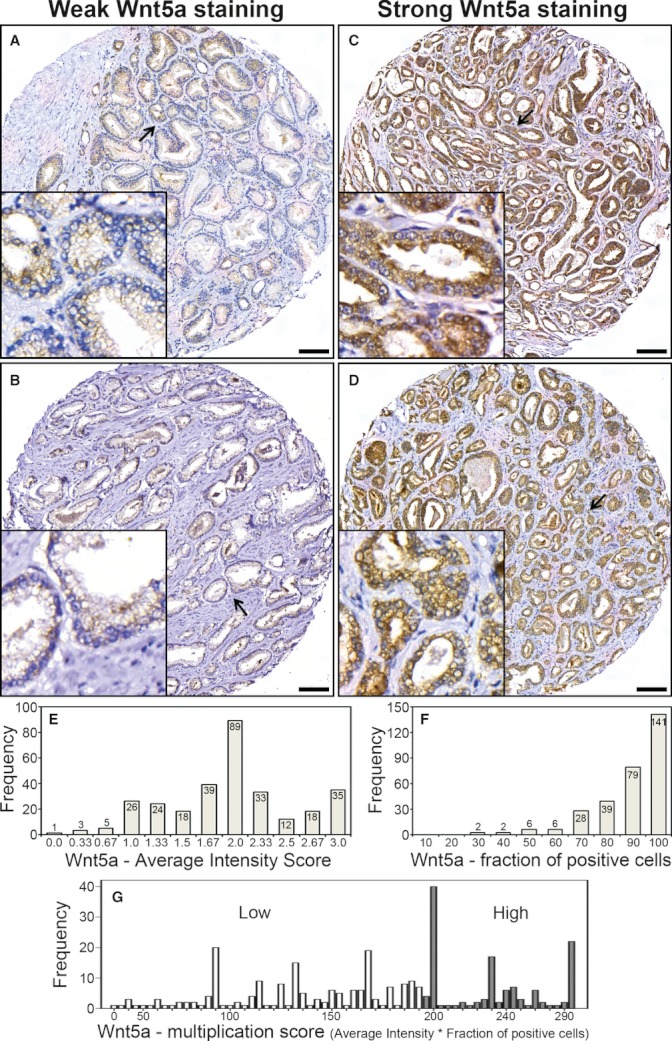
Wnt5a immunostaining in prostate cancer. (A) and (B) illustrate representative examples of weak Wnt5a protein expression, whereas (C) and (D) show strong Wnt5a protein expression in tissue microarray cores of primary prostate tumors obtained after radical prostatectomy. All inserts in the panels represent higher magnifications of selected areas (arrows) (bars 100 μm). (E) and (F) are histograms showing Wnt5a staining average intensity score (scale 0–3) and fraction of Wnt5a-positive cells (scale 10–100), respectively. In (G), distribution Wnt5a expression is illustrated as low or high multiplication score (average intensity score × fraction of Wnt5a-positive cells) from 0 to 300 with a cut-off at 195 as identified by classification and regression tree analysis.

Wnt5a immunostaining was performed as described earlier [18] using an in-house affinity purified rabbit polyclonal anti-Wnt5a IgG preparation. This antibody is well characterized by peptide antigen and recombinant Wnt5a blocking experiments and immunohistochemistry (IHC) and Western blot of PCa cells after silencing down Wnt5a expression by siRNA [10, 18]. The cores were scored independently by a pathologist (L. H.) and one of the other authors (S. K.). Disagreements while scoring, where present, were sorted out after re-examining the cores and a consensus was reached by open discussion. For Wnt5a cytoplasmic staining, intensity as well as percentage of positive cells (fraction) were recorded. Each core was scored based on the staining intensity as follows: 0 (no staining), 1 (weak staining), 2 (moderate staining), or 3 (strong staining). Percentage of positive cells (0, 5, 10, 20, 30, …, 100%) was then multiplied by the predominant staining intensity score to get a value (“multiplication score”) for each core, ranging from 0 to 300 (Fig. 1G; Glaessgen et al. [21]. Since cancer cores from each patient were prepared in triplicates, we decided to use the average value for further analyses.

### Statistical analysis

All statistical analyses were performed using SPSS version 20 (SPSS, Chicago, IL). The patient cohort was divided into two groups based on pathological Gleason score. Tumors of Gleason score 3+4 or lower were classified as low-grade cancers, whereas Gleason score 4+3 or higher represented high-grade cancers. Criteria for this classification were based on the fact that primary Gleason grade is the most important indicator to predict outcome of the disease. A Gleason score 7 represents either a 3+4 score, where pattern 3 is the primary and prevalent Gleason grade or a 4+3 score, where pattern 4 is the primary and predominant Gleason grade. The presence of high Gleason grade (4 or 5) disease has the greatest risk for the patients compared to a low Gleason grade (3 or less) disease. Previous studies have shown that within Gleason score 7, patients with Gleason 4+3 have three times increased risk of developing lethal PCa compared to patients with Gleason 3+4 [22, 23]. Classification and regression tree analysis was used to find optimal cutoffs for dichotomization of the material into Wnt5a subgroups of prognostic value in relation to BCR [24, 25]. Kaplan–Meier curves were created to demonstrate BCR-free survival (outcome) and log rank (Mantel–Cox) test was used to compare BCR-free survival between different Wnt5a expression groups. Cox regression analysis was used for estimation of hazard ratio (HR) in uni- and multivariate analysis. Fisher's exact test was used to compare categorical variables among groups. *P*-values of <0.05 were considered significant.

## Results

### Wnt5a protein tissue expression in prostate cancer

Immunostaining of the TMA for Wnt5a revealed a cytoplasmic pattern of immunoreactivity in tumor cells in accordance with our previous results. Illustrations and scoring details are given in Figure 1A–G. Patients were classified into two groups based on a cutoff value obtained by classification and regression tree analyses (see Material and Methods). The optimal cutoff value was identified at multiplication score 195. Patients were then grouped as low expression/weak staining (with score 0–195) and high expression/strong staining (196–300). Of the 312 patients, tumor cores from nine patients were either lost or were damaged and not able to score, leaving 303 patients for scoring. We were able to score triplicate cores from 202 (67%) of 303 patients and duplicate cores from 79 (26%) patients, and average values were reported for these patients. High-Wnt5a protein expression was observed in nearly 41% of the cancer cases compared to 82% which we observed in our previous study [18]. This difference may be related not only to the fact that the current cohort included a larger proportion of high-grade cancers more but also to the fact that we used an average score as the current TMA contained tumor cores in triplicate, which was not the case in our previous study. Wnt5a protein expression in relation to Gleason score was also investigated (Table 2). In low-grade cancers (Gleason 3+4 or less) and in high-grade cancers (Gleason 4+3 or higher), the high expression of Wnt5a protein was 38% and 48%, respectively, which was not significantly different (*P* = 0.183, Fisher's exact test). Wnt5a immunostaining did not correlate with SMS, seminal vesicle invasion (SVI), extraprostatic extension (EPE) or clinical T-stage. These results are in strong agreement with our recent study.

**Table 2:** Distribution of Wnt5a protein expression and different clinico-pathological variables

		Wnt5a expression			
			
Variables	Groups	Low	High	Total	*P*- value#
All cancers		178 (59%)	125 (41%)	303	
Gleason score	Low- grade*	120 (62.5%)	72 (37.5%)	192	
	High- grade**	33 (52%)	30 (48%)	63	0.183
SMS	Negative	84 (60%)	55 (40%)	139	
	Positive	69 (59%)	47 (41%)	116	0.898
SVI	No	132 (59%)	90 (41%)	222	
	Yes	21 (64%)	12 (36%)	33	0.707
EPE	No	82 (60%)	55 (40%)	137	
	Yes	71 (60%)	47 (40%)	118	1
Clinical stage	T1	96 (59%)	68 (41%)	164	
	T2 and T3	57 (63%)	34 (37%)	91	0.594

EPE, extraprostatic extension; SMS, surgical margin status; SVI, seminal vesicle invasion.

* Low-grade PCa defined as pathological Gleason score ≤3+4.

** High-grade PCa defined as pathological Gleason score ≥4+3.

Wnt5a expression low and high (according to multiplication score; see Results).

# *P*-values as obtained using Fisher's exact test.

### Wnt5a protein expression to predict clinical outcome after radical prostatectomy

**Figure 2:**
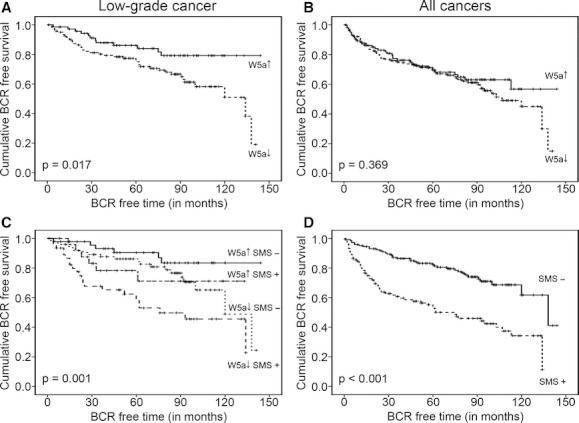
Associations between Wnt5a protein expression and/or surgical margin status (SMS; positive or negative) and BCR-free time after radical prostatectomy in PCa patients. (A) Kaplan–Meier curves plotted for high (↑) and low (↓) Wnt5a protein expression (for definition, see Results) and BCR-free time in low-grade cancers only (Gleason score ≤3+4). (B) Kaplan–Meier curves plotted between high- or low-Wnt5a protein expression and BCR-free time in the whole patient material (low-grade and high-grade cancers). (C) Kaplan–Meier curves showing the effect of Wnt5a protein expression and SMS on BCR in low-grade cancers. The curves were plotted after combining Wnt5a protein expression and SMS. (D) Kaplan–Meier curves demonstrating the association between SMS and BCR-free survival in all patients. *P*-values <0.05 indicate statistically significant differences in outcome between the most and least favorable group.

To examine if Wnt5a expression level is a significant predictor of BCR-free time after RP, Kaplan–Meier curves were plotted between strong or weak Wnt5a immunostaining and BCR-free time (Fig. 2). We had access to complete clinical follow-up data from 262 patients, of whom we had scoring data from 255 patients, leaving this number for subsequent analyses. There was no significant association between Wnt5a expression and outcome regarding the whole patient material (Fig. 2B; Table 3). When the current Stockholm cohort was dichotomized based on Gleason score, we observed that Wnt5a expression level was significantly associated with outcome in PCa patients with low-grade cancers as patients in this group with high-Wnt5a protein expression had significantly longer BCR-free time after RP compared to patients with low-Wnt5a expression (*P* = 0.017; Fig. 2A). There were no significant differences in outcome between patients with different Wnt5a expressions in high-grade cancer PCa patients (data not shown).

**Table 3:** Uni- and multivariate analyses of factors influencing BCR-free survival

	Groups	*n* (%)	HR (95% CI)	*P*-value#
All cancers – univariate analyses				
Gleason Score	Low-grade*	198 (76)	1 (Reference)	
	High-grade**	64 (24)	3.4 (2.3–5.0)	<0.0001
Wnt5a	High	102 (40)	1 (Reference)	
	Low	153 (60)	1.2 (0.8–1.8)	0.369
SMS	Negative	144 (55)	1 (Reference)	
	Positive	118 (45)	2.9 (1.9–4.4)	<0.0001
SVI	No	229 (87)	1 (Reference)	
	Yes	33 (13)	2.3 (1.4–3.6)	0.001
EPE	No	143 (55)	1 (Reference)	
	Yes	119 (45)	2.2 (1.4–3.2)	0.0001
Low-grade cancers – univariate analyses				
Wnt5a expression	High	72 (37.5)	1 (Reference)	0.017
	Low	120 (62.5)	2.1 (1.1–4.1)	
SMS	Negative	124 (63)	1 (Reference)	
	Positive	74 (37)	2.6 (1.5–4.4)	0.0002
Wnt5a and SMS	Wnt5a high SMS negative	46 (24)	1 (Reference)	
	Wnt5a low SMS Positive	46 (24)	4.9 (2.0–12)	0.001
	Wnt5a high SMS Positive	26 (14)	2.3 (0.73–7.0)	0.158
	Wnt5a low SMS negative	74 (38)	2.1 (0.83–5.2)	0.116
Low-grade cancers – multivariate analyses				
Wnt5a expression	High	72 (37.5)	1 (Reference)	0.024
	Low	120 (62.5)	2.1 (1.1–4.0)	
SMS	Negative	120 (62.5)	1 (Reference)	
	Positive	72 (37.5)	2.1 (1.2–3.7)	0.013
SVI	Yes	8 (4)	1 (Reference)	
	No	184 (96)	2.3 (1.4–3.6)	0.269
EPE	No	124 (65)	1 (Reference)	
	Yes	68 (35)	1.5 (0.8–2.8)	0.154

*n*, frequency; HR, hazard ratio; CI, confidence interval.

* Low-grade PCa defined as pathological Gleason score ≤3+4.

** High-grade PCa defined as pathological Gleason score ≥4+3.

Wnt5a expression high and low (for definition see Results).

# *P*-values as obtained by Cox regression analyses.

Gleason score, SMS, SVI, and EPE are well-established factors predicting outcome in PCa patients after RP. With regard to the whole patient cohort, univariate analyses revealed that all these parameters predict BCR-free survival, whereas in low-grade cancers high-Wnt5a expression also predicts favorable outcome (Table 3). Cox regression multivariate analysis revealed that SMS but not SVI or EPE significantly predicted outcome in low-grade cancers. When Wnt5a protein expression was added to this model, multivariate analysis showed that both Wnt5a protein expression and SMS independently predict BCR-free survival, as patients with high-Wnt5a protein expression and negative SMS have better outcome (Fig. 2C; Table 3).

## Discussion

Two recent studies, one of which was from our group, have shown contradictory results on the role of Wnt5a protein expression to predict outcome in PCa patients after RP [18, 20]. Our previous study [18] involved 503 PCa patients (Malmö TMA) classified as 84% having low-grade cancer based on Gleason score. In the present study, we validated Wnt5a protein expression as a predictive biomarker in a TMA from a completely independent cohort (Stockholm) consisting of 312 patients. In the whole patient material, we did not find any predictive value of Wnt5a protein. In our previous study, we however showed that high-Wnt5a protein expression significantly correlated with favorable outcome in terms of longer relapse-free survival [18]. The different results on outcome between the two studies may be attributed to a larger proportion of high-grade tumors (24% compared to 10%) and more frequent recurrences (38% compared to 27%) in the present cohort (Stockholm) than those in the previous one (Malmö; Table 1). When the current patient cohort was grouped into low-grade cancers and high-grade cancers based on pathological Gleason score, we found that Wnt5a protein expression was significantly associated with better outcome in PCa patients with low-grade cancers as patients in this group with high-Wnt5a protein expression had significantly longer BCR-free time after RP compared to patients with low-Wnt5a expression. In contrast to our results, Yamamoto et al. [20] suggested that patients with high/positive Wnt5a protein expression have a worse outcome compared to patients with low/negative Wnt5a protein expression. Yamamoto et al. performed their study on a smaller group of PCa patients (98) and used a somewhat different classification of cancers based on Gleason score with 24.5% classified as high-grade tumors (Gleason score ≥8). Differences in number of PCa sample studied together with the use of different antibodies may, in part, explain contradictory results on the association between Wnt5a protein expression and relapse-free survival after RP as presented by our group [18] and by Yamamoto et al. [20]. The current Stockholm TMA includes 24% high-grade tumors which is similar to the patient material described by Yamamoto et al., with 24.5% of PCa patients classified as Gleason score ≥8. Despite this similarity in proportion of low- and high-grade cancers, we could not find the same predictive ability of Wnt5a expression as they found in their study. Instead, we successfully confirmed our previous finding of a positive predictive value of Wnt5a protein expression in PCa patients and showed that high-Wnt5a protein expression significantly associated with longer relapse-free survival time in low-grade PCa. The antibody used in our studies has been well characterized and documented by peptide antigen and recombinant Wnt5a blocking experiments and IHC and Western blot of PCa cells after silencing Wnt5a expression [10, 18]. Moreover, genetic variations between the two study groups cannot be neglected since these studies have been performed on patients from different genetic ancestry. Other possible reasons for differences between the studies may be that Yamamoto et al. performed their study on whole tissue sections from prostatectomies whereas in our studies we used section from TMAs of RP specimens. While scoring we recorded both percentage of positively immunostained Wnt5a expressing tumor cells and staining intensity, and then multiplied the two to get a “multiplication score.” Classification and regression tree analysis was then used to find optimal cut-offs for dichotomization of the material into Wnt5a subgroups to determine the prognostic value in relation to BCR. However, Yamamoto et al. used only percentage of positively stained cancer cells in tumor region and described tissues as Wnt5a positive when the staining was present in more than 50% of the cancer cells.

It is well known that patients with negative SMS have a significantly longer relapse-free time after RP compared to patients with positive SMS [26]. The same was observed in our clinical material, as there was a statistically significant difference in clinical outcome between patients with negative and positive surgical margins when Kaplan–Meier curves were plotted (Fig. 2D). Interestingly, we noticed that Wnt5a expression may have an effect on outcome in patients with positive SMS. Patients with low-grade cancers, displaying high-Wnt5a protein expression and positive SMS have similar relapse-free time after RP compared to patients with low-Wnt5a staining and negative SMS (Fig. 2C; Table 3). This finding may be of importance from a therapeutic point of view and indicates an opportunity to treat PCa patients with low-grade tumors and positive SMS by targeting Wnt5a signaling. Molecular mechanism by which Wnt5a affects clinical outcome in PCa is not yet fully understood, although it is likely that Wnt5a has an effect on invasion of tumor cells in PCa, as has been described in other malignancies [18, 27, 28]. Foxy5, a Wnt5a mimicking peptide, has been shown to impair cell motility of breast cancer cell lines with low endogenous Wnt5a levels and to significantly inhibit breast tumor metastasis to lung and liver in vivo [28]. The use of Foxy5, shown to reduce invasion in PCa cell lines [18], is therefore a hypothetical future therapeutic option in PCa which deserves to be further explored.

Validation of biomarkers is a major challenge in scientific research, illustrated by the fact that only few biomarkers shown to be promising in a particular cancer have successfully been validated independently. We therefore consider our present confirmation of Wnt5a to predict clinical outcome in patients after RP for localized low-grade PCa to emphasize an important role of Wnt5a in PCa progression. PSA-based screening for PCa has led to unwanted overdetection and overtreatment of less harmful prostate cancers [29]. Evaluation of Wnt5a protein expression to identify low-grade cancers with favorable outcome may thus be useful to avoid overtreatment in this group of patients. However, this needs to be further studied in preoperative biopsies before it can be implemented in a clinical setting. In conclusion, we have now been able to confirm our previous results, that Wnt5a can be used as a predictive biomarker in PCa patients, which supports a view that Wnt5a is a future therapeutic target in patients with PCa cells displaying low endogenous expression of Wnt5a.
